# Comparative analysis of the biomechanics of anterior cervical discectomy and fusion with multiple segmental plates fixation versus single multilevel plate fixation: a finite element study

**DOI:** 10.1186/s12891-022-05796-7

**Published:** 2022-09-07

**Authors:** Weibo Huang, Ye Tian, Hongli Wang, Fei Zou, Xiaosheng Ma, Jianyuan Jiang, Ruoyu Li

**Affiliations:** Department of Orthopedics, Huashan Hospital, Fudan University, 12 Mid-Wulumuqi Road, Shanghai, 200040 China

**Keywords:** Anterior cervical discectomy and fusion, Internal fixation, Construct failure, Adjacent segment degeneration, Finite element study

## Abstract

**Background:**

This study aimed to compare the biomechanical differences between anterior cervical discectomy and fusion (ACDF) with multiple-level separate plates and conventional long plates by using finite element analysis.

**Methods:**

The following four finite element models were created to simulate various fixations: (1) C4–6 ACDF with multiple plates, (2) C4–6 ACDF with a single plate, (3) C3–6 ACDF with multiple plates, and (4) C3–6 ACDF with a single plate. The maximum Von-mises stress of the cage and fixation, compressive force of the adjacent intervertebral discs and range of motion (ROM) of different segments in the four models were calculated and analyzed.

**Results:**

For C4–6 ACDF, the maximum Von-mises stress of the cage and fixation was lower in the multiple plate fixation model in all motion states. Similarly, for the C3–6 ACDF models, the peak stress of the C3–4 and C5–6 cages was lower with multiple plates fixation in all motions but the stress of the C4–5 cage in the multiple plates model was slightly higher in flexion, bending and rotation. Besides, applying multiple plates in C3–6 ACDF models resulted in a decreased maximum stress of the fixation under different motions except for bending. In both the C4–6 ACDF and C3–6 ACDF models, the ROM values of the adjacent motion segments were lower in the multiple plates models in extension, bending and rotation. In the C4–6 ACDF models, the peak stress on the adjacent intervertebral discs in the multiple plates models was slightly smaller. In C3–6 ACDF models, the maximum stress on the adjacent intervertebral discs was larger in the single-plate model under flexion, bending and rotation movements.

**Conclusion:**

Multiple plates fixation has a positive effect on increasing stiffness and maintaining the ROM of adjacent segments, indicating lower risk of construct failure and adjacent segment degeneration. Further studies are required to confirm its efficacy in clinical practice.

## Background

Anterior cervical discectomy and fusion (ACDF) is a well-established and effective treatment for symptomatic cervical myelopathy and radiculopathy [1, 2]. However, there are issues with ACDF, particularly for multilevel ACDF [3]. An important complication is adjacent segment degeneration (ASD), which may be caused by abnormal range of motion (ROM) of the segment adjacent to the fusion level [4, 5]. Previous studies have reported an increased rate of ASD in patients with multilevel ACDF compared to that in patients with single-level ACDF [3, 6]. The result might be associated with the abnormal load distribution on the cervical spine after multilevel ACDF due to the long plate applied in the surgery [7]. Furthermore, the load sharing and transfer of stresses through these long plates to the adjacent segments might increase the risk of other complications, including construct failure, misalignment, nonunion or pseudarthrosis [8, 9]. The prominence of the long plates could also affect the surrounding tissue, causing persistent postoperative dysphagia or even esophageal injury [10, 11].

Recently, the application of separate segmental fixation for multilevel ACDF such as zero-profile spacer has been suggested by some researchers [12, 13]. It is reported that use of multiple single-level fixation possesses some biomechanical advantages such as lower risk of ASD compared to multilevel long fixation [12]. Moreover, the separate segmental fixation might be beneficial with regards to decreasing the complication associated with injury of the surrounding tissue caused by the long plate such as post-operative dysphagia [13, 14].

Previous researches have primarily focused on the biomechanical performance of zero-profile spacers, however, ACDF with multiple separate plates is a noteworthy schedule for separate segmental fixation due to decreased subsidence and better improvement in cervical lordosis when compared with zero-profile spacers [15, 16]. To best of our knowledge, few studies reported the biomechanical comparison between the fixation with multiple separate plates and with a single long plate [17, 18]. Finn et al. compared the biomechanical efficacy of applying two-level noncontiguous plates or three-level plate for patients with two noncontiguous levels requiring treatment, but their study focused on the forces exerted on the intermediate and adjacent levels [17]. Rios and Eastlack studied the stiffness of multiple segmental plates using fatigue testing. Both studies were based on cadaveric specimens and focused on three-level fusion [18]. The current study applied finite element (FE) analysis, which has been widely applied in research of spinal biomechanics to simulate the complicated construct and allows calculation of relevant values [19]. The abovementioned advantages enabled us to perform more biomechanical evaluations on both two-level and three-level fusions. The aim of this study was to compare the biomechanical differences regarding the ROM and stress of the cage, fixation and adjacent discs between ACDF with multiple-level separate plates and with continuous long plates using FE analysis.

## Methods

### Establishment of the intact cervical model

Computed tomography (CT) images of a 27-year-old male subject without a history of cervical spine disease were collected from the Department of Radiology at our hospital. This study was approved by the Institutional Review Board of our hospital. The slice thickness was 0.625 mm, and the images from C2 to C7 were exported as the Digital Imaging and Communications in Medicine format. The images were then imported into Mimics software to generate a three-dimensional model. Subsequently, the three-dimensional model was imported into Geomagic Studio 2013 (3D Systems, Inc., Rock Hill, South Carolina, USA) for further modification, including polishing and mesh. The ‘Construct Patches’ and ‘Grid and Fit Surfaces’ tools were applied during the procedure and the modified model was subsequently exported as stereolithography (STL) format. The STL file was imported into SolidWorks 2017 computer-aided design software (Dassault Systèmes SolidWorks Corporation, Waltham, Massachusetts, USA). The cortical bone, cancellous bone, endplate, annulus fibrosus, nucleus pulposus, articular cartilage, and other elements were created based on previous literature [20]. An cervical spine model of C2-C7 was created with all the elements mentioned above. The model created in SolidWorks 2017 CAD was imported into the ANSYS Workbench 2020 software for mesh and calculation (ANSYS, Ltd., Canonsburg, Pennsylvania, USA). The detailed parameters of the cortical bone, cancellous bone, endplates, annulus fibrosus, nucleus pulposus and other properties were defined based on previous studies and are listed in Table 1 [12, 19]. Given that the ligaments generally did not result in obvious resistance force against the physiological compression, the ligaments were set as ‘tension only’ spring element to simulate this feature and the biomechanical parameters were set as Table 2 showed [19].

**Table 1:** Mechanical parameters of applied component

Component	Young’s modulus (MPa)	Poisson’s ratio	Reference
Cortical bone	12,000	0.3	[12, 19]
Cancellous bone	450	0.29	[12, 19]
Facet joint cartilage	10.4	0.4	[12]
Endplate	500	0.4	[12]
Nucleus	1	0.49	[12, 19]
Annulus fibrosus	50	0.45	[19]
PEEK	3000	0.3	[12]
Bone graft	450	0.29	[12]
Titanium alloy	110,000	0.3	[12, 19]

**Table 2:** Ligaments force-displacement behavior (F: N, dl: mm)

Ligament	Parameter	Value						
**ALL**								
	**F**	0.0	5.5	10.0	13.5	16.5	19.5	54.5
	**dl**	0.0	1.2	2.5	3.7	4.8	6.0	20.0
**PLL**								
	**F**	0.0	4.5	5.5	11.0	13.5	15.0	47.0
	**dl**	0.0	1.2	2.2	3.2	4.3	5.0	20.0
**LF**								
	**F**	0.0	1.5	3.0	3.5	5.0	5.5	11.0
	**dl**	0.0	1.8	3.5	5.1	6.9	8.0	20.0
**ISL&SSL**								
	**F**	0.0	1.5	2.0	4.0	5.0	5.5	9.8
	**dl**	0.0	1.3	2.8	4.1	5.5	7.0	20.0
**CL**								
	**F**	0.0	1.5	2.6	4.3	5.2	5.4	10.5
	**dl**	0.0	3.6	5.0	7.5	9.5	9.9	20.0

*ALL* Anterior longitudinal ligament, *PLL* Posterior longitudinal ligament, *LF* Ligamentum flavum, *ISL* Interspinous ligament, *SSL* Supraspinous ligament, *CL* Capsular ligaments

### Boundary and loading conditions

Previous studies were referenced to set the boundary and loading conditions for biomechanical testing [21–24]. The contact type between the screws, plates, and vertebrae was set as the connection mode [23, 24]. Nodes of the inferior surface of the C7 vertebra were constrained. The type of contact between the intervertebral discs and adjacent endplates was defined as the bonded mode. The contact type of the facet joint was set as frictional and the friction coefficient was 0.1 [21, 22]. The frictionless contact type was set between the cancellous bone filling the cage and the vertebra. The ‘bonded mode’ contact type was applied between the cage and the vertebra.

Subsequently, a follower load of 73.6 N combined with 1.0 N·m bending moments of flexion, extension, lateral bending, and axial rotation were applied on the superior surface of the C2 vertebra to simulate spinal motions [25, 26]. The ROM of the model was recorded and compared with previous studies for validation of the model [27, 28].

### Surgical simulation

**Fig. 1:**
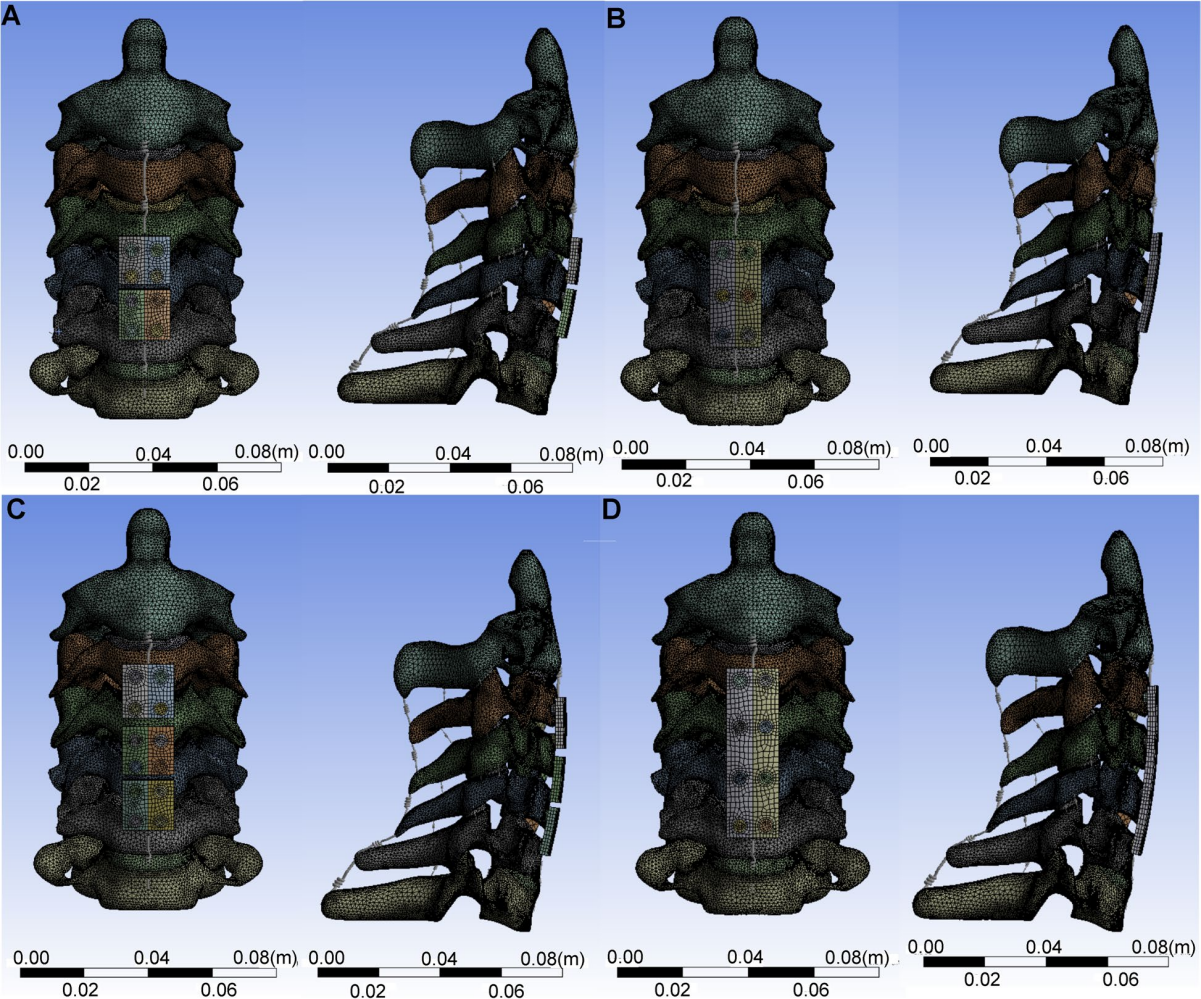
Scheme of various fixation constructs created in the study. **A** C4–6 ACDF with segmental plate fixation model (C4–6 multiple plates model); **B** C4–6 ACDF with long plate fixation model (C4–6 single plate model); **C** C3–6 ACDF with segmental plate fixation model (C3–6 multiple plates model); **D** C3–6 ACDF with long plate fixation model (C3–6 single plate model). MP = multiple plates SP = single plates

Models of the plates, cages and screws applied in the current study were created in SolidWorks 2017 CAD. The cages were 14 mm length, 12 mm width, and 6 mm height. The screws used were 14 mm long and 4 mm in outer diameter. The length of the plates used in the experiment was selected with 14 mm width and 2.5 mm thickness. The annulus fibrosus, nucleus pulposus, endplates and anterior longitudinal ligament were removed at the surgical segments. To simulate the clinical strategy of preventing ASD, plates were installed to enable the plate ends to be inferior to one-third of the superior vertebrae or superior to one-third of the inferior vertebrae along the disc. Four models of the cervical spine with various fixations were constructed in the current study. The models included: (1) C4–6 ACDF with segmental plate fixation (C4–6 multiple plates model); (2) C4–6 ACDF with long plate fixation (C4–6 single plate model); (3) C3–6 ACDF with segmental plate fixation (C3–6 multiple plates model); and (4) C3–6 ACDF with long plate fixation (C3–6 single plate model). The schemes for the different fixations are shown in Fig. 1.

### Data collection

Combining the created models with a 73.6 N follower load and 1.0 N·m bending moments of different motions, the calculation was performed. Several parameters were recorded to evaluate the biomechanical performance of the various fixation plans as follows: (1) the maximum Von-mises stress of the cage,(2) the maximum Von-mises stress of the plates and screws, (3) the compressive force of the adjacent intervertebral discs (IVD), and (4) the ROM of the spine segment from C2–7. For the bending and rotation movements, the acquired data for the left and right bending/rotation were averaged in this study. The mesh convergence test was performed during the procedures concerning the Von-mises stress to decrease potential errors caused by the mesh size. The areas where the stress was concentrated were checked and the element sizes of the areas were altered and refined. With increasing mesh size, the results that showed an almost stable solution with a variability of less than 5% were recognized as acceptable and recorded in this study.

## Results

### Model validation

**Fig. 2:**
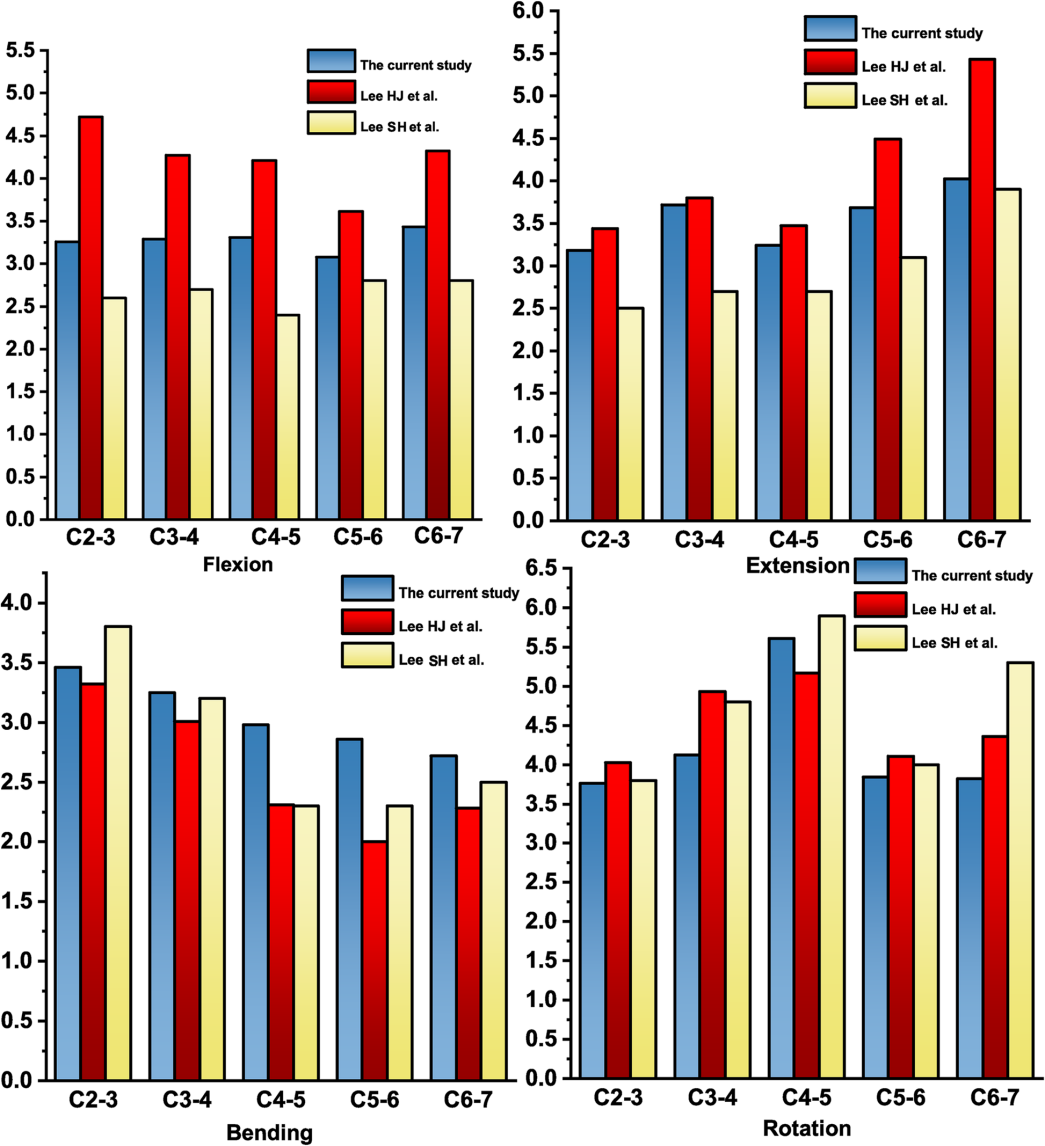
Model Validation

For validation, the ROM of the intact model was compared with that of previous studies [27]. The ROM of the intact C2–7 model in various motion states was listed in Table 3 and was close to that of previous biomechanical studies, as shown in Fig. 2 [27, 28].

**Table 3:** The range of motion of the intact cervical spine at different segments (°)

	Flexion	Extension	Bending	Rotation
C2–3	3.26	3.18	3.46	3.76
C3–4	3.29	3.72	3.25	4.12
C4–5	3.31	3.24	2.98	5.61
C5–6	3.08	3.68	2.86	3.84
C6–7	3.43	4.02	2.72	3.82

### Loading of the cage and fixation

**Fig. 3:**
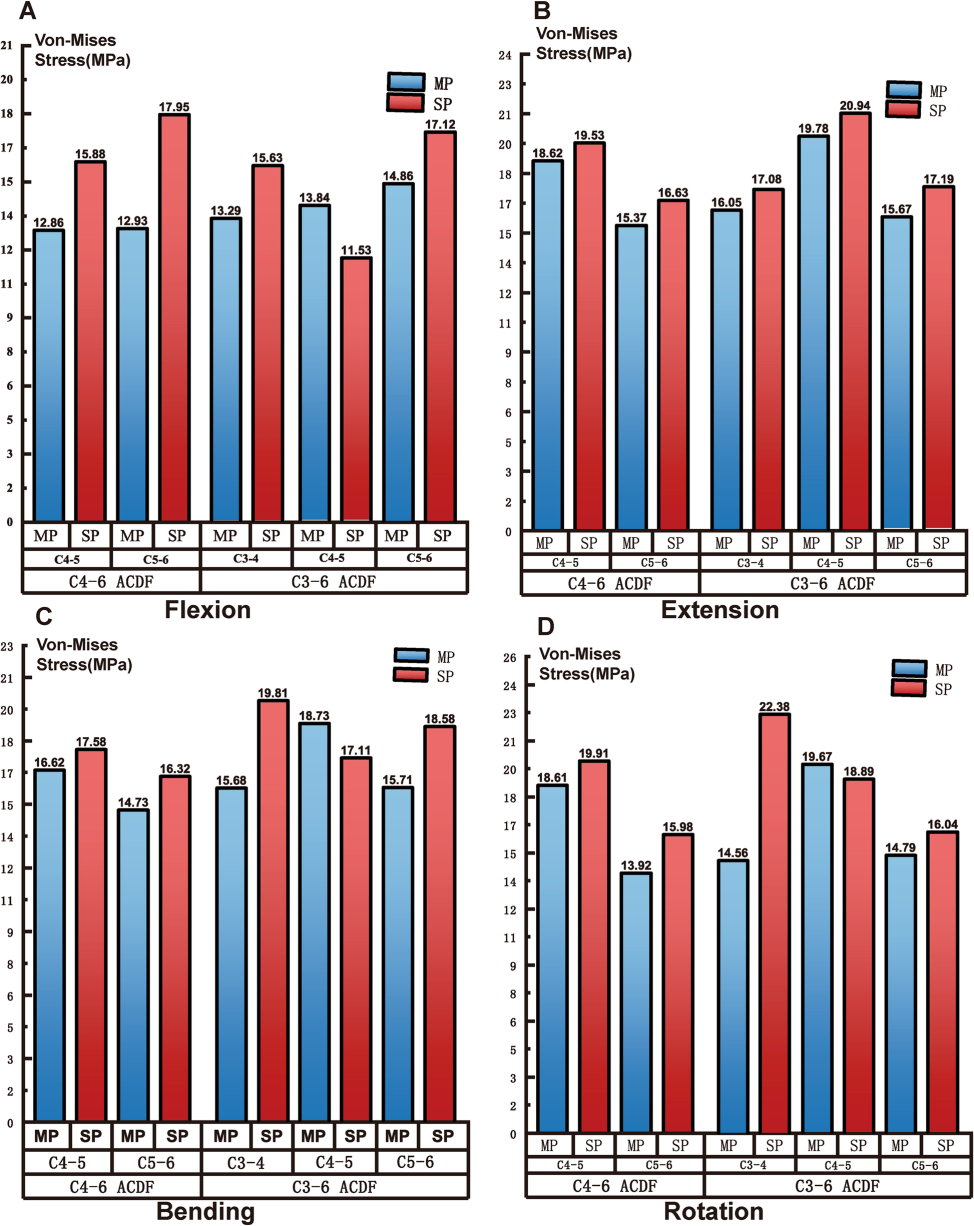
The maximum Von-mises tress of the cage. MP = multiple plates SP = single plates

The maximum Von-mises stress of the cage is presented in Table 4 and Fig. 3. As the figure shows, for the C4–6 ACDF models, the maximum stress of both cages in the C4–6 multiple plates model was lower in the multiple plate fixation model than in the single plate model in all motion states. Similarly, for the C3–6 ACDF models, the peak stress of the C3–4 and C5–6 cages was lower with the multiple plates fixation in all motions. However, the stress of the C4–5 cage in the multiple plates model was slightly higher than that of the single plate model in flexion, bending and rotation.

**Table 4:** The maximum Von-mises stress of the cage (MPa)

		Flexion	Extension	Bending	Rotation
**C4–6 ACDF**					
	C4–5 MP Cage	12.86	18.62	16.62	18.61
	C4–5 SP Cage	15.88	19.53	17.58	19.91
	C5–6 MP Cage	12.93	15.37	14.73	13.92
	C5–6 SP Cage	17.95	16.63	16.32	15.98
**C3–6 ACDF**					
	C3–4 MP Cage	13.29	16.05	15.68	14.56
	C3–4 SP Cage	15.63	17.082	19.81	22.38
	C4–5 MP Cage	13.84	19.78	18.73	19.67
	C4–5 SP Cage	11.53	20.94	17.11	18.89
	C5–6 MP Cage	14.86	15.67	15.71	14.79
	C5–6 SP Cage	17.12	17.19	18.58	16.04

*MP* multiple plates, *SP* single plate

**Fig. 4:**
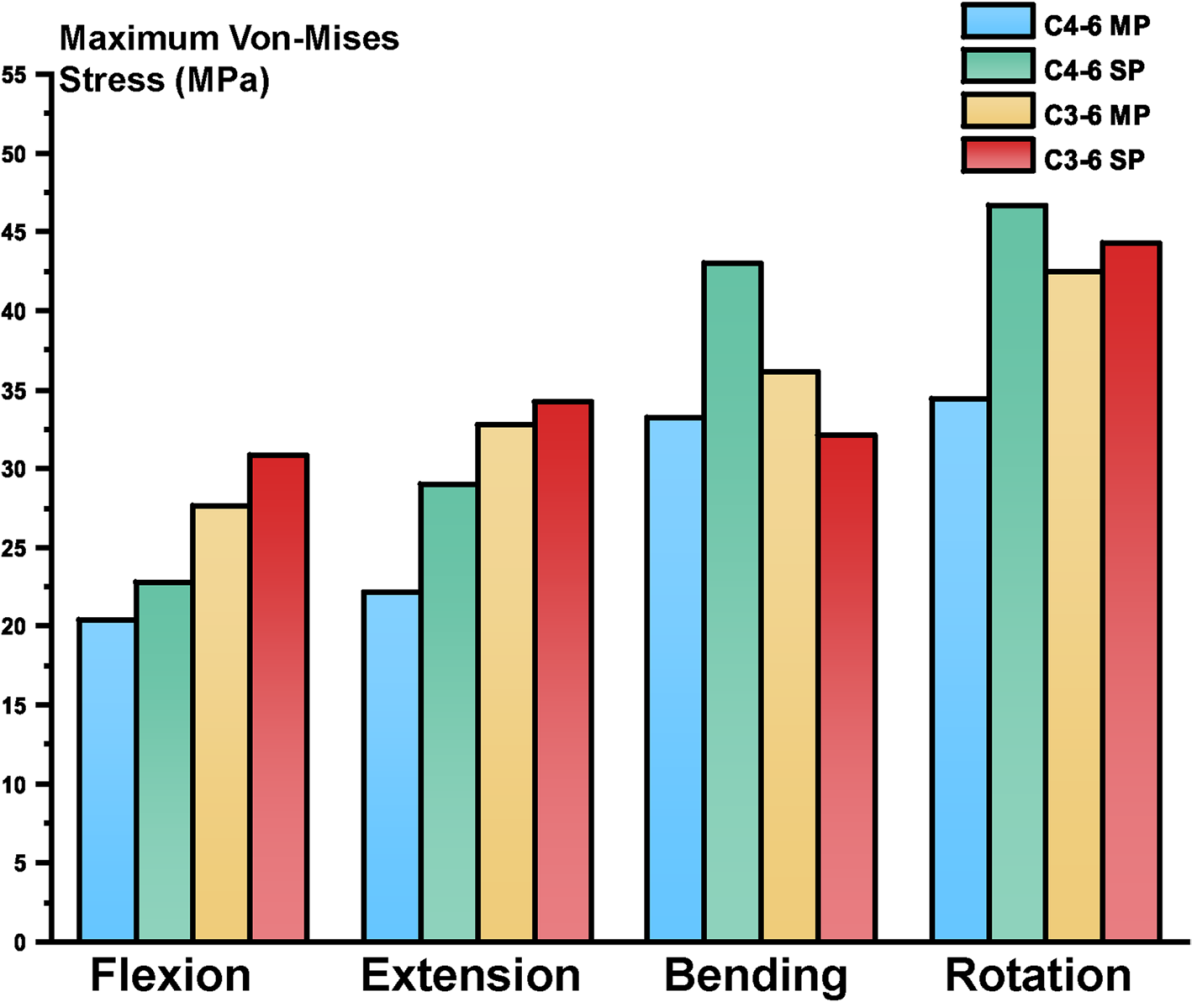
The maximum loading of the plates and screws. MP = multiple plates SP = single plates

As Fig. 4 presents, the maximum loading of the plates and screws in the C4–6 ACDF models resembles the stress of the cage (Table 5). The multiple plates model afforded a lower peak stress than the single model in all motion states. In the C3–6 ACDF models, applying multiple plates led to lower maximum stress of the fixation under different simulation motions except for the bending movement.

**Table 5:** The maximum loading of the plates and screws (MPa)

	Flexion	Extension	Bending	Rotation
C4–6 ACDF MP	20.38	22.76	27.68	30.88
C4–6 ACDF SP	22.15	28.98	32.73	34.21
C3–6 ACDF MP	33.18	43.04	36.18	32.08
C3–6 ACDF SP	34.40	46.65	42.49	44.31

*MP* multiple plates, *SP* single plate

### ROM of different segment

**Fig. 5:**
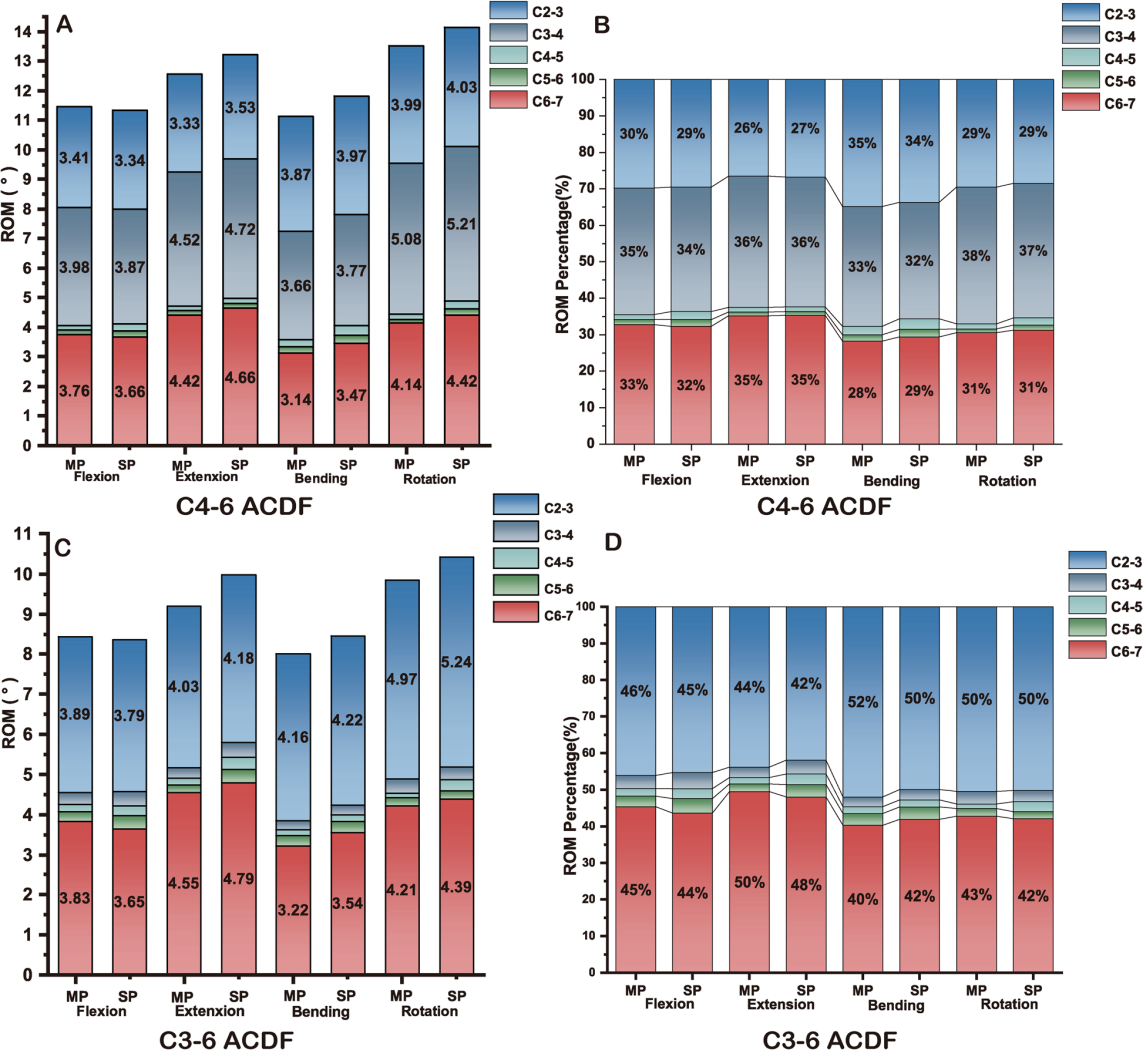
The value and percentage of ROM for different cervical segments from C2–7. MP = multiple plates SP = single plates

**Fig. 6:**
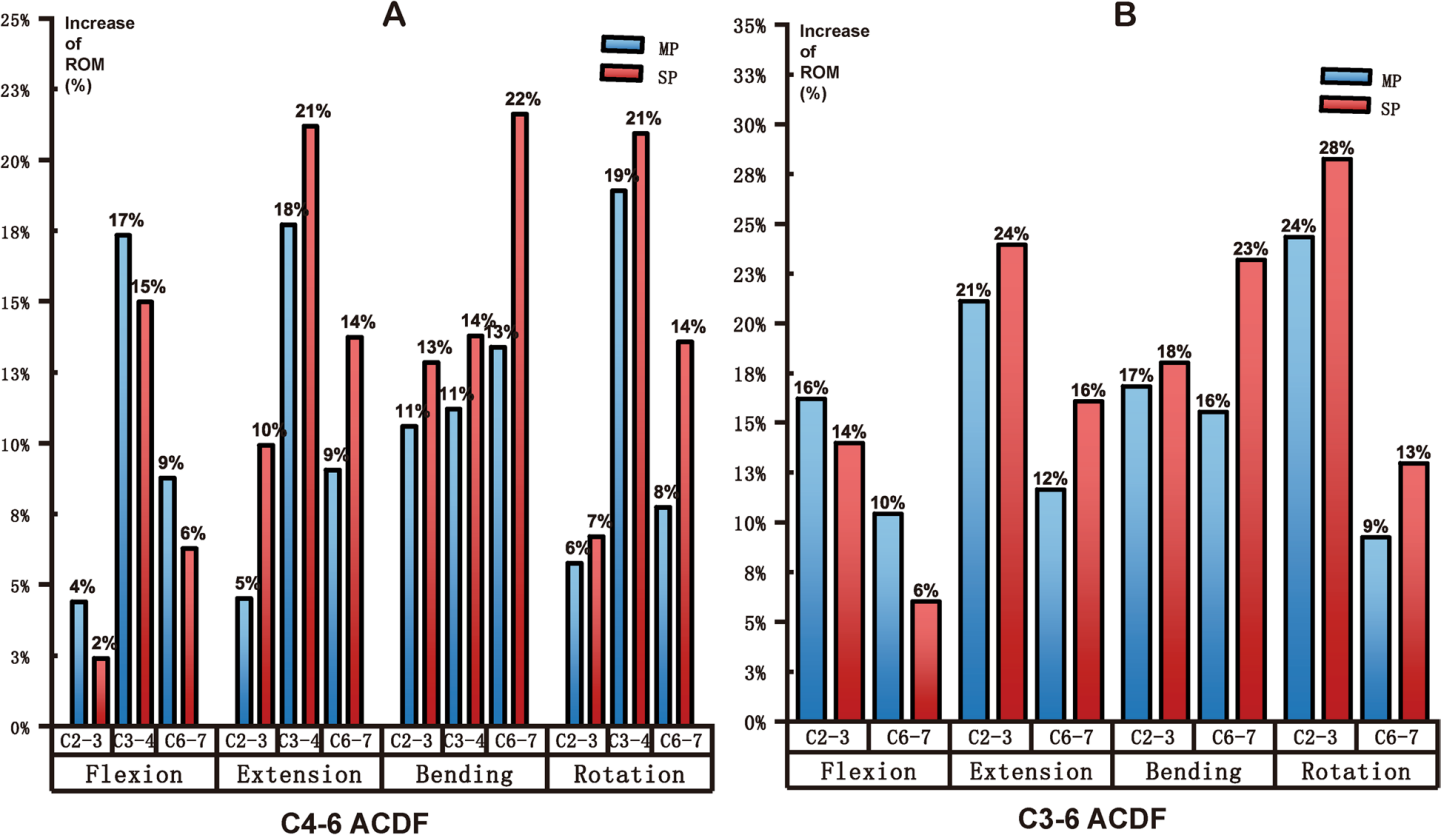
Increase of ROM of adjacent segments in various motion states. MP = multiple plates SP = single plates

Tables 6, 7 and Fig. 5 present the values and percentages of ROM for the different cervical segments from C2–7. Given that the ROM values of the fused levels were almost zero, the total ROM values of both models decreased but the unfused segments increased to some extent in all ACDF models compared to the intact cervical spine model. It could be noted that no obvious divergence between the multiple plates fixation and single plate fixation could be found regarding the percentage of ROM in both C4–6 and C3–6 ACDF models in various motion states. However, the ROM values differ, especially in the bending and rotation states. For both C4–6 ACDF and C3–6 ACDF models, in the extension, bending and rotation conditions, the ROM values of the superior and inferior adjacent motion segments were lower in the multiple plates models. To be more detailed, Fig. 6 and Table 8 present the increase of ROM of different segments in various motion states for the models involved in the study. For C4–6 fusion surgery, the increase in ROM ranged between 4% and 19% in multiple plates fixation models and between 2 and 22% in single plates fixation models. During flexion, the ROM increase of C2–3, C3–4, and C6–7 was larger in the multiple plates fixation models, but opposite results were found in the three other movements. A similar trend was observed in the C3–6 fusion models.

**Table 6:** The value and percentage of ROM for different cervical segments from C2–7 in C4–6 ACDF models (°)

	Flexion	Extension	Bending	Rotation
MP C2–3	3.41 (29.76%)	3.33 (26.49%)	3.87 (34.80%)	3.99 (29.49%)
SP C2–3	3.34 (29.48)	3.53 (26.70%)	3.97 (33.64%)	4.03 (28.50%)
MP C3–4	3.98 (34.73%)	4.52 (35.96%)	3.66 (32.91%)	5.08 (37.55%)
SP C3–4	3.87 (34.16%)	4.72 (35.70%)	3.77 (31.95%)	5.21 (36.85%)
MP C4–5	0.16 (1.40%)	0.16 (1.27%)	0.26 (2.34%)	0.18 (1.33%)
SP C4–5	0.25 (2.21%)	0.17 (1.29%)	0.34 (2.88%)	0.28 (1.98%)
MP C5–6	0.15 (1.31%)	0.14 (1.11%)	0.19 (1.71%)	0.14 (1.03%)
SP C5–6	0.21 (1.85%)	0.14 (1.06%)	0.25 (2.12%)	0.2 (1.41%)
MP C6–7	3.76 (32.81%)	4.42 (35.16%)	3.14 (28.24%)	4.14 (30.60%)
SP C6–7	3.66 (32.30%)	4.66 (35.25%)	3.47 (29.41%)	4.42 (31.26%)

*MP* multiple plates, *SP* single plate

**Table 7:** The value and percentage of ROM for different cervical segments from C2–7 in C3–6 ACDF models (°)

	Flexion	Extension	Bending	Rotation
MP C2–3	3.89 (46.09%)	4.03 (43.85%)	4.16 (52.00%)	4.97 (50.46%)
SP C2–3	3.79 (45.28%)	4.18 (41.88%)	4.22 (49.94%)	5.24 (50.24%)
MP C3–4	0.30 (3.55%)	0.26 (2.83%)	0.21 (2.63%)	0.34 (3.45%)
SP C3–4	0.37 (4.42%)	0.38 (3.81%)	0.24 (2.84%)	0.32 (3.07%)
MP C4–5	0.18 (2.13%)	0.16 (1.74%)	0.15 (1.89%)	0.12 (1.22%)
SP C4–5	0.23 (2.75%)	0.29 (2.91%)	0.16 (1.88%)	0.28 (2.68%)
MP C5–6	0.24 (2.84%)	0.19 (2.07%)	0.26 (3.25%)	0.21 (2.13%)
SP C5–6	0.33 (3.94%)	0.34 (3.41%)	0.29 (3.43%)	0.2 (1.92%)
MP C6–7	3.83 (45.38%)	4.55 (49.51%)	3.22 (40.25%)	4.21 (42.74%)
SP C6–7	3.65 (43.61%)	4.79 (48.00%)	3.54 (41.89%)	4.39 (42.09%)

*MP* multiple plates, *SP* single plate

**Table 8:** Increase of ROM of adjacent segments in various motion states(%)

		Flexion	Extension	Bending	Rotation
**C4–6 ACDF**					
	C2–3 MP	4.40%	4.51%	10.59%	5.76%
	C2–3 SP	2.40%	9.92%	12.85%	6.70%
	C3–4 MP	17.34%	17.70%	11.20%	18.90%
	C3–4 SP	14.99%	21.19%	13.79%	20.92%
	C6–7 MP	8.78%	9.05%	13.38%	7.73%
	C6–7 SP	6.28%	13.73%	21.61%	13.58%
**C3–6 ACDF**					
	C2–3 MP	16.20%	21.09%	16.83%	24.35%
	C2–3 SP	13.98%	23.92%	18.01%	28.24%
	C6–7 MP	10.44%	11.65%	15.53%	9.26%
	C6–7 SP	6.03%	16.08%	23.16%	12.98%

*MP* multiple plates, *SP* single plate

### Loading of the adjacent disc

**Fig. 7:**
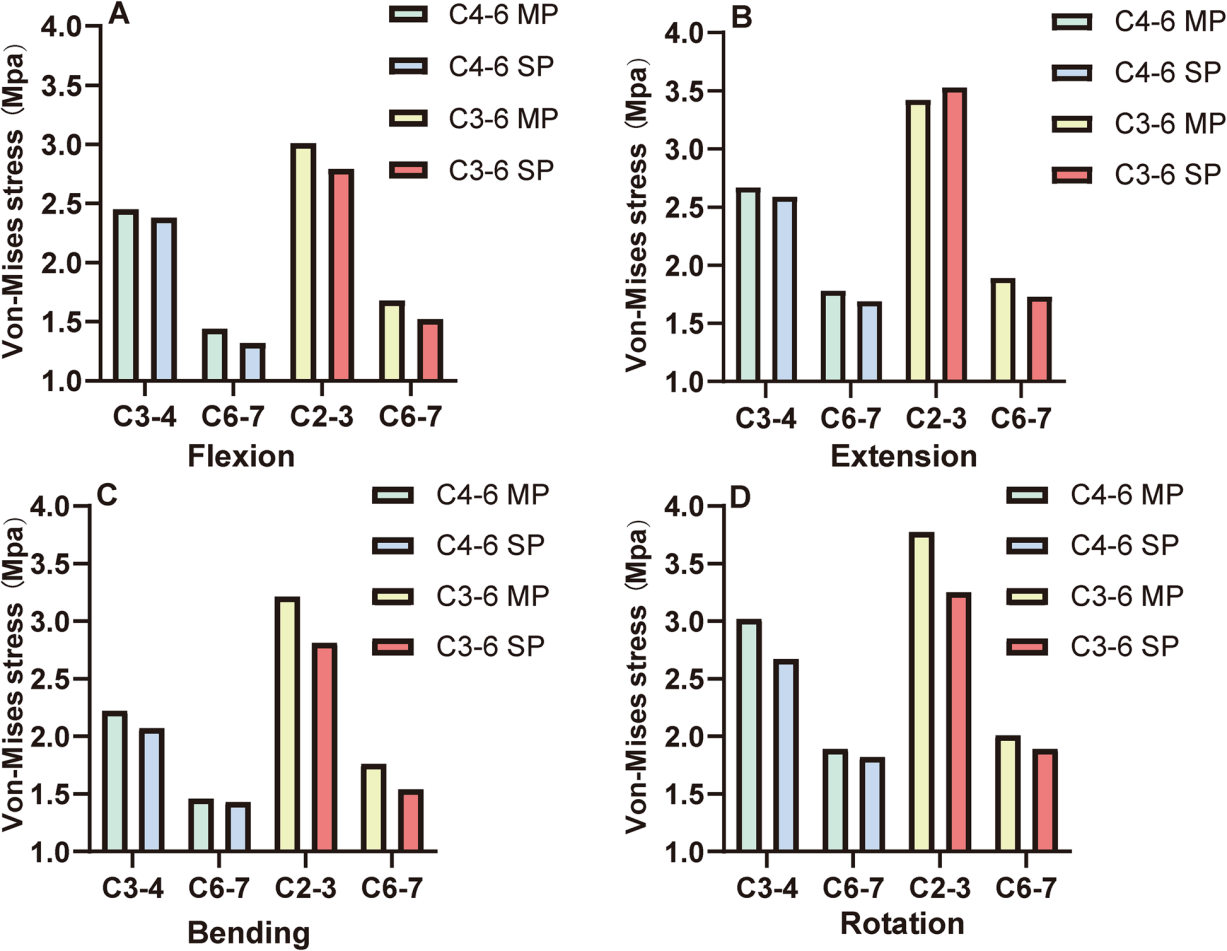
The peak stress of the intervertebral discs of adjacent segments in various motion states. MP = multiple plates SP = single plate

The stress distribution of the adjacent disc was analyzed and displayed on Table 9 and Fig. 7. Two main findings were found: (1) In C4–6 ACDF models, although the maximum stress of the adjacent IVD was close between the multiple plates fixation and single plate fixation, the peak stress of the multiple plates models was slightly smaller. In C3–6 ACDF models, the maximum stress of the adjacent IVD was larger in the single plate model under flexion, bending and rotation movements. (2) The maximum stress of the superior adjacent IVD was higher than that of the inferior adjacent IVD in all models and in all motion states.

**Table 9:** The peak stress of the intervertebral discs of adjacent segments in various motion states (MPa)

		Flexion	Extension	Bending	Rotation
**C4–6 ACDF**					
	MP C3–4 IVD	2.45	2.67	2.22	3.02
	SP C3–4 IVD	2.38	2.59	2.07	2.67
	MP C6–7 IVD	1.44	1.78	1.46	1.89
	SP C6–7 IVD	1.32	1.69	1.43	1.82
**C3–6 ACDF**					
	MP C2–3 IVD	3.01	3.42	3.21	3.77
	SP C2–3 IVD	2.79	3.53	2.81	3.25
	MP C6–7 IVD	1.68	1.89	1.76	2.01
	SP C6–7 IVD	1.52	1.73	1.54	1.89

*MP* multiple plates, *SP* single plate, *IVD* intervertebral disc

## Discussion

ACDF has been reported to be effective for the treatment of CSM, particularly in cases with severe disc degeneration due to its efficiency with regards to decompression of the spinal cord and reconstruction of cervical spine stabilization [1, 2]. The overall rate of complications varied between 13.2 and 19.3% despite the satisfying stiffness and high fusion [29]. According to previous literature, dysphagia was the most common and immediate complication after surgery, occurring in 1.7 to 67% cases [30–32]. Although the majority of patients with post-operative dysphagia could recover within 3 months, 3–35% of patients would suffer sustained dysphagia and face a risk of impairment in nutrition and quality of life [30, 31]. Some factors were considered to potentially be associated with the dysphagia in previous studies, including numbers of levels involved, operation time, the size and type of plates, and the extent of tissue dissection and retraction [10, 33, 34]. For obese patients or patients with a short thick neck, surgeons might encounter difficulties installing the single multi-level plates ideally and lead to increased surgery duration time as well as more tissue dissection and retraction, and thus would face a higher risk of dysphagia and esophageal injury. The segmental fixation schedule could be considered to decrease the risk with similar stiffness when sufficient exposure could hardly be achieved in some cases.

Previous studies reported a 0.1–0.9% incidence of mechanical failure after ACDF surgery [32]. Selecting proper fixation instruments is critical for better outcomes in patients undergoing ACDF with osteoporosis, considering that osteoporosis is regarded as a risk factor for construct failure including screw pull-out and cage subsidence [35, 36]. The maximum Von-mises stress of the internal fixation indicated that in most situations the peak stress of the plates and stress is lower in multiple plates models. In other words, the risks of fatigue and failure were theoretically lower in the model. These results could be explained by the use of more screws to share the stress applied in the model. Meanwhile, the results showed that the multiple plates model had better biomechanical performance owing to the lower peak loading of the cage. The extent of subsidence is considered to be directly associated with the high stress concentration and discrepancy in the elastic modulus between the contact interfaces. Subsidence has become a concern given that it can result in the cervical misalignment and even breakage or pull-out of the screw [9, 37]. For patients with osteoporosis, the difference between the endplates and cage would increase as the elastic modulus of the endplates decrease. In the current study, the maximum stress of the cage was higher in the single plate fixation model. One reasonable explanation for this finding is that, compared with the single plate model, the multiple plates fixation provided more screws which could share the loading and then decrease the stress of the cage. Lower maximum cage stress was associated with a lower risk of cage subsidence. Moreover, previous studies have reported that osteoporosis is associated with a slower and less reliable fusion progress [38, 39]. In clinical practice, spine surgeons would face a dilemma when surgical treatment was required for patients with bone mass loss. In this situation, the multiple plates fixation with more screws combined with appropriate medications can be considered as one of the solutions.

Adjacent segment disease has been another problem with much concern. Increasing clinical and radiologic evidence has proved accelerated degeneration process of spinal segments adjacent to the fusion level [5, 27, 40]. A long-term (5–30 years) study including 166 patients reported that worse degenerative changes were found in over 90% patients who underwent ACDF. A similar percentage (>90%) of patients with radiographic ASD was also found in another retrospective study of 177 patients receiving ACDF treatment [40]. Among the patients involved, clinical ASD was found in 19.2, and 7% of the total patients required revision surgery. Several factors have been proven to be associated with ASD. It has been demonstrated that the ROM of the adjacent segment would increase to compensate for the sacrificed ROM at the fused segment [3]. In the current study, the ROM of the adjacent segments was lower in the multiple plates fixation in most simulation situations. In other words, the ROM did not indicate a higher risk of ASD in the multiple plates fixation models. In addition, the load distribution of the adjacent disc has been considered as another factor that was related to the occurrence of ASD, and some studies regarded it as a more important factor [3, 7]. Given that the nutrition acquirement of the IVD mainly relies on the diffusion and osmosis, the nutrition exchange might be affected by the continuous overload of IVD, leading to the acceleration of IVD degeneration [41]. Similar to the findings concerning ROM, the current study found that the maximum stress of the multiple plates fixation was lower compared to the single plate fixation in most situations. Thus, the multiple plates fixation might have better biomedical performance in preventing ASD. An explanation for this result might be the load sharing by more screws, which could decrease the loading of adjacent IVDs.

Despite the aforementioned advantages, some deficits of the multiple plates could not be ignored. First, although the multiple plates procedure might require less installation time when ACDF involving three to four segments was performed without satisfying the surgical field, the surgical procedure could be more complicated and lead to longer operative time if limited segments required fixation or the exposure was not difficult. Meanwhile, the stiffness increased as more screws were applied, but the risk of complications associated with screws input such as endplates fracture and posterior vertebral walls penetration would increase. And the medical cost would be higher for patients, considering that more plates and screws were used during the surgery. However, for patients with osteoporosis or other factors leading to a higher risk of construct failure, the multiple plates fixation could be taken into account for surgeons to balance the cost and the risk for revision surgery.

The current study has some limitations. As previous studies described, the major limitation of FE analysis is model simplification [24, 42, 43]. In other words, some acceptable idealized simulations were applied. The boundary and loading conditions are more complicated under actual in vivo condition. For example, the contact type between the screws, plates and vertebrae was set as the connection mode without micromotion but the situation could rarely be fully achieved, especially in the immediate period after ACDF. Although some settings such as the ‘tension only’ spring have been applied for better simulation, the idealized element could not perfectly simulate the biomechanical reactions of the ligaments. To make different models comparable and to avoid potential confounders, the parameters were set equally in all models to make the results more convincing. Another limitation was that the model was created based on the CT DICOM files from an asymptomatic volunteer without degeneration. As previously discussed, the simulation might perfectly reflect the actual clinical scenario but the study aimed to provide a trend and reference for future studies by taking advantage of FE analysis [43].

## Conclusion

The FE analysis in the current study indicated a better biomechanical performance of the ACDF models with multiple plates fixation with regards to the stiffness and maintenance of the ROM in adjacent segments. These advantages are associated with a lower risk of related complications including construct failure and ASD. Meanwhile, the low-profile construct with multiple plates could possibility result in less injury to surrounding tissue, leading to a lower risk of post-operative dysphagia. The fixation schedule can be considered in some cases, such as in patients with osteoporosis.

## Data Availability

The datasets generated and/or analysed during the current study are not publicly available due to originality and individual privacy but are available from the corresponding author on reasonable request.

## Abbreviations

ACDF: Anterior cervical discectomy and fusio
ROM: Range of motion
CT: Computed tomography
IVD: Intervertebral disc
ALL: Anterior longitudinal ligament
PLL: Posterior longitudinal ligament
LF: Ligamentum flavum
ISL: Interspinous ligament
SSL: Supraspinous ligament
CL: Capsular ligaments
MP: Multiple plates
SP: Single plate
STL: Stereolithography
